# Recent Advances in Fault Diagnosis and Opacity Analysis in Discrete Event Systems

**DOI:** 10.3390/s26041144

**Published:** 2026-02-10

**Authors:** Agostino Marcello Mangini, Ruotian Liu, Wei Duan, Shu Zhang, Maria Pia Fanti

**Affiliations:** 1 Department of Electrical and Information Engineering (DEI), Polytechnic University of Bari, 70126 Bari, Italywei.duan@poliba.it (W.D.); mariapia.fanti@poliba.it (M.P.F.); 2 School of Electro-Mechanical Engineering, Xidian University, Xi’an 710071, China

**Keywords:** discrete event system, fault diagnosis, opacity

## Abstract

This paper continues the historical and technical trajectory of fault diagnosis and opacity analysis in discrete event systems (DESs). Whereas the previous work reviewed the foundational developments of event diagnosis and opacity, this survey focuses on recent advances over the past decade by addressing modern challenges such as communication losses, delays, distributed architectures, and cyber-attack scenarios. Specifically, we present a structured overview of diagnosability verification and enforcement across automata, Petri nets, and other DES models under these scenarios. In parallel, we review contemporary results on opacity verification and enforcement, including complexity findings, reduction techniques, and robust or attack-resilient formulations. In addition, this survey provides an updated picture of the evolving research landscape and highlights emerging themes and open problems in diagnosis and opacity for DESs.

## 1. Introduction

Discrete event systems (DESs) have provided a fundamental modeling framework for reasoning about logical behavior, coordination, and information flow in networked systems. As cyber–physical infrastructures, autonomous vehicles, robotic platforms, and large-scale networked control architectures grow in complexity, understanding what can or cannot be inferred from limited observations becomes increasingly critical. Two major research themes have emerged around this issue: *fault diagnosis*, i.e., to reveal hidden faults (usually assumed to be unobservable events) fast and accurately, and *opacity*, i.e., to conceal confidential behavior from an external observer. Although they originate from different practical concerns, i.e., safety and reliability in the case of diagnosis and security and privacy in the case of opacity, their theoretical foundations are deeply intertwined. Both rely on partial observations, both model inference through state estimators or state-based structures, and both reason about how information propagates through system dynamics [1].

Over the past decades, extensive survey papers have documented the evolution of fault diagnosis in DESs [2,3,4], reflecting both the theoretical maturity of the field and its growing practical relevance. Fault diagnosis and diagnosability analysis [5,6] form the foundation for monitoring and ensuring the safe operation of complex infrastructures such as automated manufacturing systems [7,8] and urban transportation networks. In such systems, sensing capabilities are often constrained by cost, placement limitations, or physical inaccessibility. As a result, only partial observations of system behavior are available, and certain components may remain completely sensorless. While sensorless designs can help protect critical modules from malicious tampering, faults occurring in these components may lead to severe consequences if not detected in time. This tension between limited observability and the need for timely fault localization has driven much of the research in fault diagnosis. Related work on fault identification, which aims at reconstructing DES models for fault detection, addresses a complementary problem and is therefore not covered in depth in this survey [9,10,11,12].

Diagnosability, in this context, is the system property that determines whether the occurrence of a fault can always be detected within a finite delay based solely on observable outputs. Classical diagnosis methods [13,14] aim to infer hidden faults from observed event sequences, while diagnosability verification assesses whether the system structure guarantees such inference in principle. Beyond verification, diagnosability enforcement seeks to modify or constrain system behavior so that faults become detectable, even when uncertainties or malicious behaviors are present.

Recent developments have broadened the notion of diagnosability to accommodate different fault specifications. For instance, the authors of [15] revisit repeated fault diagnosability and propose a diagnoser-based algorithm for verifying k-diagnosability. The work in [16] extends classical approaches to event-pattern diagnosability and reduces the problem to checking a linear-time property over a time Petri net via model-checking techniques. These extensions illustrate the increasing expressive power required to capture complex fault behaviors in modern applications. In contrast to the classical supervisory control methods or the approaches that incorporate timing information to prevent deadlocks [17,18,19,20,21,22,23,24,25], a different line of work introduces quiescent information [26,27,28]. Building on this idea, the authors of [29] generalize the classical notion of diagnosability in labeled Petri nets (LPNs) to systems that may contain potential deadlocks.

Meanwhile, diagnosability has been reconsidered in the presence of adversarial manipulation [30,31,32]. Attackers may attempt to conceal faults by altering or fabricating sensor readings. While many studies assume that attackers have access to the same observations as operators, real-world adversaries typically observe or compromise only part of the system. As a result, their perception of what has been successfully hidden may differ from the actual information available to an operator or monitoring algorithm. This motivates a richer investigation of diagnosis under partial, asymmetric, or corrupted information flows, linking the topic naturally to the broader theme of opacity analysis and DES-based information-security modeling. In addition, diagnosability has also been investigated under other sources of uncertainty, such as communication delays, packet losses, and intermittent observation failures, which further complicate fault inference in networked and cyber–physical systems.

Opacity was initially formalized as a qualitative indistinguishability requirement for finite-state automata: an intruder observing a system through a projection map must never be able to determine that a secret state or behavior has occurred [33,34,35,36]. Foundational works introduced and analyzed current-state opacity (CSO), initial-state opacity (ISO), initial-and-final-state opacity (IFSO), as well as language-based opacity (LBO) and various *K*-step and infinite-step variants [33,35,36,37,38,39,40].

Over the past two decades, opacity has been extended far beyond the finite-automaton setting. Petri-net models allow explicit representation of concurrency, synchronization, and resource constraints and have led to observer-like, basis-reachability-graph, and verifier-net-based verification techniques for CSO, ISO, and LBO [41,42,43]. Timed DES further augment transitions with timing information, enabling the study of timed current-state opacity, opaque time, and related notions in timed stochastic DES and time LPNs [44,45,46,47]. Networked DESs explicitly incorporate communication delays, packet losses, and reordering, providing a natural framework to reason about opacity under realistic communication imperfections and networked supervisory structures [48,49,50,51]. Stochastic DES, Markovian and probabilistic automata, and fuzzy or approximate models bring in randomness and graded uncertainty, leading to probabilistic, fuzzy, approximate, and metric-based opacity notions that quantify rather than merely decide information leakage [44,52,53,54,55].

In parallel with these modeling advances, opacity verification has developed into a technically rich area spanning automata, Petri nets, timed models, and probabilistic or fuzzy systems. Classical observer-based constructions for finite automata remain central [33,35,36,37], while Petri-net approaches rely on basis reachability graphs, verifier nets, and structural or algebraic characterizations [41,42,43]. Timed Petri nets require abstractions such as marking-class graphs, whereas probabilistic opacity is analyzed via belief-state dynamics and Markov chain semantics [44,52,53].

Beyond verification, a substantial body of work now focuses on *opacity enforcement*: the synthesis of mechanisms that modify the plant behavior or its observation channel so that opacity is guaranteed. Channel-based methods use edit and insertion functions to alter the observation stream, injecting fictitious or suppressed events so that secret executions remain indistinguishable from at least one nonsecret counterpart [56,57,58,59,60,61,62,63,64,65,66]. Control-based approaches instead synthesize supervisors that disable controllable events leading to secret-revealing behaviors [67,68,69,70,71,72]. These ideas have been extended to decentralized settings with multiple supervisors or intruders [48,49,50,69,73] and to non-logical opacity frameworks that combine secrecy with probabilities, fuzziness, or strategic information release [44,52,53,54,55,74,75].

Taken together, this survey offers a unified, cross-model perspective on opacity and its relationship to diagnosis. By integrating foundational definitions, methodological developments, and emerging frontiers across DES theory, we aim to provide researchers with a coherent view of how inference, knowledge, and information flow shape the behavior, security, and privacy of DES.

Although this survey focuses on formal models and theoretical developments, the reviewed results are strongly motivated by practical challenges arising in industrial automation and cyber–physical systems. Fault diagnosis frameworks directly address monitoring and fault isolation problems in automated manufacturing systems, robotic production lines, transportation networks, and networked control architectures, where partial observability, sensor placement constraints, and fault criticality are inherent design limitations. Likewise, opacity analysis provides a formal foundation for protecting sensitive operational information in Industrial Control Systems (ICSs) and smart grids, where revealing internal modes or system configurations may expose vulnerabilities to adversaries. Throughout this paper, the presented theoretical constructs are therefore interpreted as abstractions of real engineering constraints.

**Figure 1:**
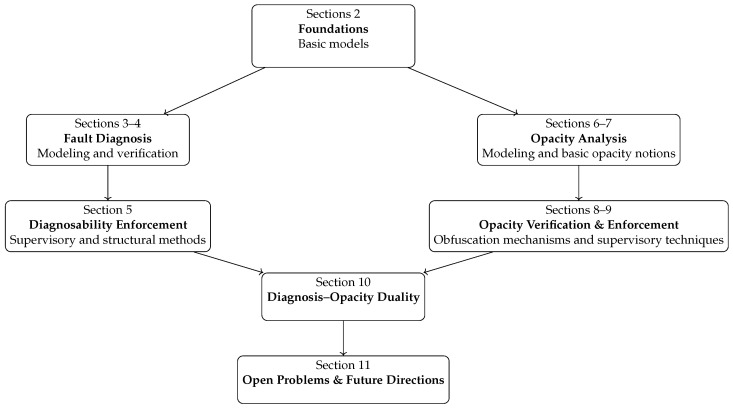
Technical roadmap of the survey.

The remainder of this survey is organized to guide the reader from foundational concepts to advanced developments and emerging challenges. Section 2 reviews the necessary preliminaries on finite-state automata and Petri nets, establishing the modeling and observation frameworks used throughout the paper. In Section 3, we present a comprehensive overview of fault diagnosis, covering modeling assumptions, classical approaches, and recent extensions under uncertainty and adversarial settings. Section 4 focuses on diagnosability verification, including classical criteria, complexity results, and verification techniques developed for systems with communication losses, delays, or attacks. Section 5 then discusses diagnosability enforcement, examining how supervisory control, event relabeling, and timing regulations can be used to ensure diagnosability in both untimed and timed DES models. Section 6 introduces the modeling foundations of opacity, including system models, observation structures, and intruder knowledge representations, and situates these elements within the broader DES literature. Section 7 formalizes the principal opacity notions, clarifying the distinction between logical opacity properties and their non-logical, quantitative extensions. Section 8 reviews verification methods across automata, Petri nets, and timed and stochastic models, highlighting how classical observer-based approaches have evolved into symbolic, structural, and probabilistic techniques. Section 9 surveys enforcement mechanisms, ranging from channel-based editing to supervisory control and distributed enforcement in networked DES, with emphasis on both logical and quantitative opacity. Section 10 examines the conceptual and methodological duality between opacity and fault diagnosis, showing how tools developed for revealing faults can be reinterpreted for concealing secrets. Finally, Section 11 outlines open problems and future research directions, including scalable verification, decentralized and networked enforcement, quantitative secrecy, and the integration of opacity with other security and performance objectives. A technical roadmap of the survey is provided in Figure 1.

## 2. Preliminaries

Let Σ denote a finite event set, and let $\Sigma^{*}$ be the set of all finite strings over Σ, including the empty string ε. For any string $\lambda \in \Sigma^{*}$, |λ| denotes its length, with |ε|=0. A language is a subset $L\subseteq \Sigma^{*}$. For $u,v\in \Sigma^{*}$, the concatenation is written as uv.

### 2.1. Automaton

A system is modeled as a finite-state automaton [6] $G=(X,\Sigma ,f,x_{0})$, where *X* is a finite set of states, Σ is a finite set of events, f:X×Σ→X is the (partial) transition function, and $x_{0}\in X$ is the initial state. The transition function extends to strings $\lambda \in \Sigma^{*}$ in the usual manner [6,76]. The language generated by *G* is defined as $L\left(G\right)=\{\lambda \in \Sigma^{*}|f\left(x_{0},\lambda \right)$ is defined}.

To characterize partial observation of a system *G*, its event set Σ is partitioned into the subset of observable events $E_{o}$ and that of unobservable events $\Sigma_{uo}$, i.e., $\Sigma =\Sigma_{o}\cup \Sigma_{uo}$. The natural projection that captures the observations generated by *G* is defined as $P:\Sigma^{*}\to \Sigma_{o}^{*}$, where P(ε)=ε, P(α)=α if $\alpha \in E_{o}$, P(α)=ε if $\alpha \in \Sigma_{uo}$, and P(λα)=P(λ)P(α) for all $\lambda \in \Sigma^{*}$ and α∈Σ.

### 2.2. Petri Net

A Petri net [5] is a quadruple N=(P,T,Pre,Post), where *P* (resp. *T*) is a finite and non-empty set of places (resp. transitions), graphically represented by circles (resp. bars) with P∩T=∅. Pre: P×T→N and Post: P×T→N are the pre- and post-incidence functions that specify the arcs directed from places to transitions, and transitions to places, respectively, where N={0,1,2,…} is the set of non-negative integers. C=Post−Pre is called the incidence matrix of a Petri net N.

A marking of a Petri net is a mapping M:P→N, and M(p) presents the number of tokens in *p* at marking *M*. 〈N, $M_{0}\rangle$ is called a net system with an initial marking $M_{0}$. Let n=|T| be the cardinality of set *T* and $T^{*}$ be the Kleene closure of transition set *T*. A transition *t* is enabled at a marking *M* if for all p∈P M(p)≥Pre(p,t) holds. An enabled transition *t* can fire, yielding a marking $M^{\prime }=M+C\left(\cdot ,t\right)$, denoted by $M[t\rangle M^{\prime }$. Given a transition sequence $\sigma =t_{1}t_{2}\cdots t_{n}\in T^{*}$, σ is said to be enabled at a marking *M* if there exist markings $M_{1}$, $M_{2}$, …, $M_{n}$ such that $M[t_{1}\rangle M_{1}[t_{2}\rangle M_{2}\ldots M_{n-1}[t_{n}\rangle M_{n}$ holds, denoted by $M[\sigma \rangle M_{n}$. In this case, $M_{n}$ is said to be reachable from *M*. Write M[σ〉 if $M_{n}$ is of no interest. The set of all markings reachable from $M_{0}$, denoted by $R(N,M_{0})$, defines the reachability set of $\langle N,M_{0}\rangle$, i.e., $R\left(N,M_{0}\right)=\left\{M\in N^{\left|P\right|}|\exists \sigma \in T^{*}:\;M_{0}[\sigma \rangle M\right\}$.

From a sensing perspective, partial observation provides an abstract representation of practical sensing constraints in DESs. Observable event sets correspond to sensor outputs, logged signals, or communicated measurements, whereas unobservable events model sensing blind spots, limited measurement resolution intermittent sensor availability.

## 3. Fault Diagnosis

Fault diagnosis in DESs was first formally introduced in [77,78,79], where the authors established a model-based framework for detecting and identifying faults based on partially observed event sequences. Since, in practical applications, sensors are typically limited and only a subset of events can be observed, DES models naturally operate under partial observation. This observation constraint motivates the need for diagnosis algorithms capable of inferring fault occurrences from observable behaviors. Over the past decades, fault diagnosis has evolved into a rich research area encompassing various modeling frameworks and increasingly complex system specifications. In the following parts, we provide a structured overview of fault diagnosis approaches across different DES models. We begin with classical automaton-based methods, which form the theoretical foundation of diagnosability analysis. We then discuss fault diagnosis for bounded and unbounded Petri nets, where concurrency and resource-sharing introduce new computational challenges.

### 3.1. Automaton Based Fault Diagnosis

Fault diagnosis in DESs seeks to determine whether an unobservable fault has occurred based on the partial information generated by the system. In the automaton framework, the problem was first formalized in [13], where the system behavior is described by a finite-state automaton and observations correspond to projections of event sequences onto the set of observable events. A fault is modeled as an unobservable event whose execution alters the system state. The goal of diagnosis is to decide, after a finite number of subsequent observations, whether such a fault has taken place. Research on online fault diagnosis in automata-based DESs develops systematic procedures that use partial observation to infer hidden events [13,77,78]. A common approach constructs an estimator structure that tracks the set of all states compatible with the observation sequence. When all states in this estimate correspond either exclusively to faulty behavior or exclusively to non-faulty behavior, the system is considered diagnosable at that point. This estimator forms the foundation for many verification methods.

Another widely studied method introduces a diagnoser automaton, which evolves deterministically with the observation sequence and explicitly records whether the fault status is certain, uncertain, or normal. The diagnoser allows the identification of cycles in which the fault status remains ambiguous. Such cycles signal a violation of diagnosability because they represent observation-consistent behaviors in which the system may alternate indefinitely between faulty and non-faulty states without revealing the true status. Identifying the existence or absence of such cycles provides a practical criterion for diagnosability verification.

In the presence of the attacks, Kang et al. [80] introduce the stealthy joint diagnoser, which reveals how an attacker can mislead or obscure fault diagnosis and can also be used for online diagnosis under attacks. Lin et al. [81] propose the cyber-attack diagnoser (CA-diagnoser), which addresses diagnosability verification in the presence of attacks and supports online diagnostic decision making based on the constructed structure.

### 3.2. Petri Net Based Fault Diagnosis

Petri nets (PNs) provide a compact and expressive modeling framework for capturing concurrency, causality, and resource constraints in DESs. Due to their ability to represent complex industrial processes more naturally than automata, a substantial body of work has focused on fault diagnosis in PN and labeled PN (LPN) models. Existing diagnostic techniques can be broadly categorized into (i) ILP-based methods, which formulate the online diagnosis problem as an integer linear programming problem without enumerating the entire reachable state space, and (ii) structure-based methods, which rely on the construction of specialized diagnosers or abstractions derived from the underlying PN structure. This subsection reviews these two main methodological directions and summarizes their recent advances.

#### 3.2.1. ILP

Several studies have addressed the fault diagnosis problem for Petri nets (PNs) using optimization-based techniques. The work in [14] introduces a marking abstraction method that enables the diagnosis problem to be efficiently analyzed. Building on this idea, an online diagnosis scheme based on integer linear programming (ILP) is proposed in [82]. This ILP-based method is later extended to labeled Petri nets (LPNs) in [83], allowing multiple transitions to share the same observable label. Further improvements are reported in [84], where the approach in [83] is optimized to yield a more computationally efficient online diagnosis algorithm for LPNs. Unlike graphical or structure-based approaches, another line of research avoids constructing reachability graphs or enumerating markings. Instead, online diagnosis is achieved by solving ILP problems [82,83,84,85] or by performing state-estimation–based computations [86,87,88]. These methods are typically more scalable for large or unbounded PNs. A more recent study [89] investigates diagnosis under a specific class of cyber attacks, namely replacement–removal attacks, which delete or manipulate sensor readings. Such attacks may cause existing ILP-based diagnosers [83,84] to produce incorrect decisions. To address this issue, ref. [89] strengthens both the structural components and reasoning mechanisms of the diagnoser. Even under worst-case attack scenarios, the proposed augmented diagnoser guarantees that the diagnosis outcome remains uncertain rather than incorrect. The method applies to both bounded and unbounded LPNs and provides diagnosis results that explicitly reflect the uncertainty introduced by the attack.

#### 3.2.2. Structure-Based Techniques

Another major direction relies on structure-based techniques. Using the basis reachability diagnoser, ref. [90] proposes an online fault diagnosis approach for LPNs based on a compact representation of the reachable basis markings. In the context of networked DESs, ref. [91] studies robust fault diagnosis for LPN-modeled systems subject to communication delays and losses. The notion of networked diagnosability is introduced to characterize whether every fault can be identified after a bounded number of observations. To solve the diagnosis problem, a networked basis diagnoser is developed, together with a necessary and sufficient condition for verifying networked diagnosability. Other works incorporate adversarial behaviors into the diagnosis framework.

### 3.3. Other Models

**Figure 2:**
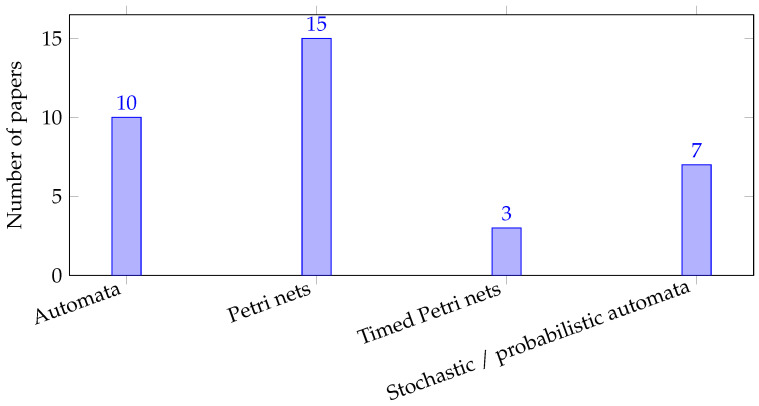
Distribution of fault diagnosis-related publications by primary modeling formalism.

Beyond automata- and Petri net-based approaches, several studies investigate fault diagnosis problems within alternative modeling frameworks. Researchers have examined decentralized architectures [92,93], in which multiple local diagnosers with partial observations cooperate to infer fault occurrences. In distributed settings, coordination among diagnosers is achieved through information exchange or consensus mechanisms, as explored in [94,95]. In particular, ref. [95] proposes a distributed diagnosis method based on iterative set-intersection refinements, enabling local components to collaboratively narrow down the set of possible system states. Fault diagnosis has also been extended to timed DESs, where timing constraints and clock information play crucial roles in characterizing diagnosability [96]. Moreover, stochastic DES models have been utilized to handle uncertainty in event occurrences and system behaviors, leading to probabilistic formulations of diagnosability and robust diagnosis strategies [97,98]. These alternative models broaden the applicability of fault diagnosis techniques and address practical challenges inherent in large-scale, uncertain, or time-sensitive systems. Building on recent advances in fluid-model representations [99,100], one may further explore how such scalable frameworks can be extended to address fault diagnosis while avoiding the large state space of discrete models. Figure 2 shows that fault diagnosis studies predominantly rely on Petri net models and automata, while timed Petri nets remain comparatively underrepresented. This suggests a promising research direction in developing fault diagnosis approaches for timed Petri nets and other expressive models.

In contrast to DESs with regular languages, fault diagnosis problems are also solved in unbounded Petri nets that may generate nonregular languages [101,102,103,104].

## 4. Diagnosability Verification

Diagnosability verification is a fundamental problem in the area of fault-tolerant DESs. It concerns determining whether every fault occurring in a system can be detected within a finite number of observable events, despite partial observation. In other words, diagnosability ensures that faults cannot remain permanently ambiguous when only limited sensor information is available. This section reviews several major lines of research on diagnosability verification. We begin with the classical diagnosability analysis framework, which provides the foundational definitions and verification methods. We then discuss diagnosability under communication imperfections, such as observation losses and delays, followed by diagnosability in adversarial environments, where attackers intentionally manipulate observations. These settings extend the classical theory to more realistic and challenging scenarios commonly encountered in networked and cyber–physical systems.

As diagnosability analysis grows more sophisticated across different modeling frameworks, computational complexity becomes an important consideration. The work in [105] provides a systematic study of the complexity of diagnosability (and opacity) verification for Petri nets. The results show that, in the presence of concurrency and unboundedness, diagnosability verification may require extremely high computational resources, revealing intrinsic limitations of brute-force state space exploration. These findings highlight the necessity of scalable analysis techniques, especially for large or structurally complex systems.

To mitigate this complexity, several model simplification strategies have been proposed. The study in [106] introduces a set of reduction rules for LPNs that allow the removal of certain unobservable transitions and specific observable transitions before diagnosability analysis is performed. When the structural conditions of these rules are satisfied, the reduced model preserves diagnosability, ensuring that no essential diagnostic information is lost. These reduction rules help decrease the size of the underlying state space, yielding significant computational savings while retaining the correctness of verification results. Together, complexity analysis and reduction techniques form an important complement to the various diagnosability verification methods reviewed in this section.

In practical terms, diagnosability verification characterizes whether the available sensor information associated with event occurrences is sufficient to distinguish faulty from non-faulty behavior within a bounded delay. Diagnosers and verifiers can thus be viewed as logical monitoring layers built on top of sensor streams, whose effectiveness depends on sensor placement and reliability.

### 4.1. Classic Diagnosability

Diagnosability verification for DESs under ideal conditions, meaning no observation losses, delays, or adversarial attacks, has been extensively studied using two main approaches: integer linear programming (ILP) and graph-based analysis. The ILP-based method in [107] formulates diagnosability verification as an optimization problem. Although effective, solving ILP problems is known to be NP-hard, which limits scalability for large systems. The majority of classical works rely on graph-based analysis. The diagnoser approach, introduced in [77,78,79,108], constructs an auxiliary automaton that tracks the evolution of the system under partial observation. A DES is diagnosable if and only if its diagnoser contains no indeterminate cycles. However, computing the diagnoser requires exploring the entire state space, yielding exponential complexity with respect to the system size. To reduce computational effort, verifier-based approaches were proposed in [79,108,109], where the verification task can be performed in polynomial time with respect to the state dimension, providing a significantly more scalable alternative. Besides ILP and diagnoser techniques, formal verification methods have also been applied. The work in [110] encodes diagnosability as a linear temporal logic (LTL) formula over transition systems and verifies it using model-checking tools such as SPIN and NuSMV. Comparative experiments highlight the competitiveness of model-checking-based verification relative to DES-specific tools like DESLab and Supremica. More recently, ref. [111] proposed a unified framework for verifying several observational properties—including diagnosability—in partially observed DESs, further consolidating the theoretical foundations of classic diagnosability analysis. Some works [26,112] study the problem of asynchronous diagnosability enforcement in DESs based on supervisory control theory. In addition, several studies have investigated diagnosability verification in time-dependent models. Specifically, ref. [113] addresses the verification of codiagnosability for DESs modeled by constant-time automata, while ref. [114] focuses on pattern diagnosability.

### 4.2. Under Loss and Delay

In many practical networked DESs, communication between sensors, measurement sites, and diagnosers may suffer from packet losses, transmission delays, or temporary disconnections. Such imperfections can compromise classical diagnosability and motivate the study of robust diagnosability and networked codiagnosability. The work in [115] examines weak diagnosability under both communication delays and packet losses. It shows that while delays do not affect weak diagnosability, packet losses can negatively impact the ability to determine fault occurrences. In decentralized networked systems subject to delays and intermittent losses, ref. [116,117] introduce the notion of network codiagnosability, providing necessary and sufficient conditions for ensuring that faults can be detected by at least one local diagnoser despite unreliable communication. Intermittent communication failures that lead to observation loss are systematically studied in [118], which develops a robust diagnosability framework for LPNs. Similar problems involving transient sensor failures are considered in [119,120]. Timing aspects are incorporated in [121], which proposes a timed NDES model with maximal communication delays and intermittent losses. The timed model is then converted into an equivalent untimed structure for verification. More recently, ref. [122] proposed a delay-resilient diagnosis method using sequence numbers to mitigate the ambiguous effects caused by delays in networked DESs. Beyond delays and losses, sensor unreliability may arise from nondeterministic readings due to noise or partial failures. The study in [123] introduces a uniform diagnosability framework based on LTL over infinite traces, capable of addressing a broad class of unreliable sensor behaviors. The work in [124] formalizes and studies diagnosability verification and enforcement in LPNs under observation and control delays.

### 4.3. Under Attack

In cyber–physical systems, adversaries may intentionally manipulate sensor readings or communication channels to obscure fault occurrences. Diagnosability verification under such attack scenarios has recently become an important research direction. Considering multiple attackers with limited observation capabilities, Lin et al. [81] developed a cyber-attack diagnoser (CA-diagnoser) for verifying diagnosability in the presence of malicious interference. Kang et al. [32] construct a joint diagnoser that captures coordinated attack strategies capable of misleading operators or preventing correct diagnosis. A broader body of work studies the impact of sensor-reading attacks on diagnosability [30,125,126,127,128,129]. The decentralized diagnosis method in [128] ensures correct inference despite local attacks. The notion of tamper-tolerant diagnosability, introduced in [126], formalizes the requirement that faults must be detectable even under adversarial manipulation. Subsequent works [32,130,131,132] investigate attack strategies capable of concealing faults, employing specialized structures such as unfolded verifiers and stealthy joint diagnosers. Denial-of-service and deception attacks are addressed in [125] using a robust test diagnoser, while ref. [30,81] develop equivalent-automaton-based methods to detect both faults and attacks concurrently. Extensions of diagnosability under uncertainty include stochastic DESs [133] and new variants such as epistemic diagnosability [134], which concerns whether a system user can detect information leakage to an intruder within bounded delay. Pattern-based diagnosability and decentralized variants are explored in [135,136]. The work in [137] focuses on the active diagnosis for LPNs that may enter deadlocks under coordinated sensor and actuator attacks.

## 5. Diagnosability Enforcement

Diagnosability enforcement concerns modifying the behavior or structure of a discrete-event system (DES) so that the system becomes diagnosable when diagnosability is not satisfied initially. Unlike diagnosability verification, which only checks whether faults can be detected within finite delay, enforcement actively ensures that ambiguous behaviors are eliminated or restricted. Two mainstream approaches have been widely studied: supervisory control-based enforcement, where a supervisor disables or restricts certain controllable events to guarantee diagnosability, and event relabeling-based enforcement, where selected events are reassigned observable labels to enhance distinguishability between normal and faulty behaviors. These methods aim to minimally modify the system while ensuring diagnosability. In the following subsections, we separately discuss diagnosability enforcement for untimed models, where the system dynamics are governed solely by discrete event sequences, and for timed models, where timing constraints or clocks influence enforceability. Each setting introduces different challenges and solution techniques.

### 5.1. Untimed Model

In the framework of untimed DESs without attacks, the diagnosability enforcement problem is typically addressed by modifying either the observation structure or the control behavior of the system. Several works focus on adjusting the observation structure [138,139,140,141,142,143,144]. In [144], diagnosability is enforced by selecting an optimal set of observations based on a Markov decision formulation that minimizes sensing cost. In stochastic DESs, diagnosability is achieved by dynamically enabling or disabling sensors depending on the system conditions [139,140]. Other studies [141,142,143,145] introduce relabeling functions and formulate integer linear programs to enforce diagnosability when relabeling operations are associated with numerical costs. Another line of research uses supervisory control to prevent diagnosability violations by disabling specific controllable events [27,29,138,146,147]. The works in [146,147] address diagnosability enforcement for deadlock-free systems, while refs. [27,29] extend the approach to plants that may contain deadlocks. More recent developments consider systems under sensor manipulations. The study in [31] investigates enforcement under replacement, deletion, and insertion of sensor readings, thereby addressing a form of robust diagnosability under observation tampering.

### 5.2. Timed Model

In timed DESs, each observation includes not only the sequence of events but also their occurrence times. Consequently, two observation strings containing the same events may still be distinguishable if their timing information differs. This inherent expressiveness allows timing constraints to play a direct role in diagnosing faults. As a result, diagnosability enforcement in timed models often requires regulating not only the enabled behavior of the system but also the timing of controllable events. The study in [148] investigates diagnosability enforcement from the perspective of active diagnosis. When a system is not diagnosable, supervisory control can be used to prevent faults from occurring silently by appropriately restricting the system’s evolution. In the timed setting, such enforcement may involve adjusting the permissible time intervals of controllable transitions so that timing information becomes sufficiently informative to distinguish faulty and non-faulty behaviors. Motivated by this idea, ref. [148] first constructs a verifier for time-interval automata to check diagnosability. Based on the verifier, diagnosability is enforced by regulating the occurrence times of selected controllable events—by tightening their time intervals—and by disabling certain controllable events when necessary. This line of work illustrates that, in timed DESs, timing regulation becomes an additional and powerful mechanism for diagnosability enforcement, complementing traditional approaches that rely solely on event disabling, relabeling, or observation restructuring in untimed models.

### 5.3. Similar Work

Several related studies investigate problems closely connected to diagnosability enforcement, particularly focusing on how diagnosability can be violated or compromised through adversarial behaviors. Kang et al. [32] construct a joint diagnoser structure for diagnosability verification, providing insights into circumstances under which diagnosability can fail. A series of works [130,131,132] analyze diagnosability in networked DESs modeled by LPN under malicious external attacks. In particular, ref. [131] examines the opposite perspective of codiagnosability enforcement [149] and robust codiagnosability [120,123,125], studying how an attacker can intentionally violate codiagnosability. The motivation is to design attack strategies capable of concealing the occurrence of critical faults and compromising the system’s ability to diagnose them. For example, an intruder manipulating sensor readings in communication channels may prevent a security-critical system, such as a banking network, from detecting abnormal or faulty behaviors, rendering the system non-diagnosable under attack.

Table 1 highlights the fundamental trade-offs between automata-based and Petri net-based fault diagnosis frameworks. Automata-based approaches benefit from conceptual clarity and mature verification techniques but suffer from state-space explosion and limited ability to represent concurrency. Petri net-based methods provide a more faithful modeling of concurrent and resource-sharing systems and enable diagnosis of unbounded plants, at the cost of higher structural complexity. The table clarifies that no single framework dominates. Instead, method selection depends on the desired balance between modeling capability and scalability.

## 6. Opacity Analysis

**Figure 3:**
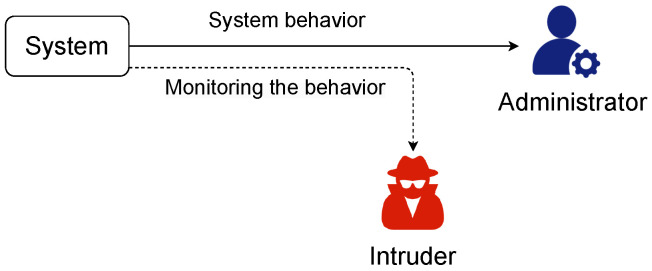
Illustration of opacity.

Opacity is an information-flow property that characterizes what an external observer (the intruder) can or cannot infer about the secret behavior of the system under partial observation, as shown in Figure 3. Any formal treatment of opacity begins with a precise specification of the following:

1. What the system does (the underlying system model);
2. What the intruder sees (the manner in which observations are generated);
3. What the intruder knows (the representation of what an intruder can infer).

This tripartite structure appears throughout the opacity literature beginning with early formulations of security and opacity in DESs [33,34] and remains the basis of modern extensions.

### 6.1. System Models

Opacity has been investigated under a wide range of modeling formalisms, reflecting the increasing complexity of modern cyber–physical systems. The dominant framework uses classical finite state automata, which form the backbone of the literature and underpin many of the foundational results on verification and enforcement of opacity [56,57,58,61,62,63,64,67,68].

Beyond finite state automata, Petri net models provide expressive representations for concurrent and resource-constrained systems. Opacity verification in this setting often relies on symbolic state-space abstractions, such as basis reachability graphs and LPNs verifiers [41,42,43], and is particularly useful when concurrency cannot be encoded compactly in automata.

Timed automata/Petri nets incorporate timing information into transitions or markings, enabling the study of opacity notions that depend on temporal patterns, which includes opaque time, time-bounded opacity, and timed current-state opacity [44,45,46,47]. In parallel, networked DES models augment the underlying automaton or Petri net representation with explicit channel states to capture communication delays, packet losses, and reordering, allowing opacity to be analyzed under realistic communication imperfections [48,49,50].

Markov chains and probabilistic automata introduce randomness into transitions, leading to probabilistic or quantitative opacity formulations that assess secrecy in terms of belief or likelihood rather than binary predicates [52,53]. Fuzzy and approximate automata generalize further by accommodating imprecision and metric-based reasoning, yielding graded opacity notions suitable for systems subject to uncertainty or approximate sensing [54,55].

**Figure 4:**
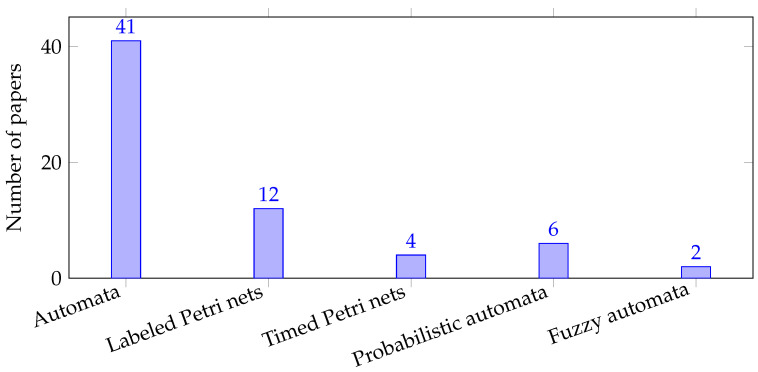
Distribution of opacity-related publications by primary modeling formalism.

Taken together, these models show how opacity theory has evolved from classical automata to a diverse family of formalisms capable of capturing concurrency, timing, communication irregularities, stochasticity, and fuzziness. Automata remain the core definitional platform, but alternative formalisms offer specialized expressive power and model-specific verification tools [1,150]. Figure 4 shows that opacity-related studies predominantly rely on automata-based models, followed by LPNs, while timed Petri nets and other uncertainty-aware formalisms remain comparatively underrepresented. This suggests a promising research direction toward extending opacity analysis to concurrency, timing, and uncertainty enriched modeling frameworks.

### 6.2. Observation Structure

The system is in general considered under partial observation, that is, the set of events is partitioned into the subset of observable events and the subset of unobservable events $\Sigma =\Sigma_{o}\cup \Sigma_{uo}$. The intruder observes only the projection $P:\Sigma^{*}\to \Sigma_{o}^{*}$. Thus, the intruder sees only the observable part of the execution; many different real traces may generate the same observation; opacity exploits this ambiguity to hide secret behavior. If the observation of the intruder were complete ($\Sigma_{o}=\Sigma$), opacity would typically be violated.

Over the years, several observation models have been considered in the literature to capture more realistic sensing and communication phenomena:

1. *Classical partial observation*: the intruder sees exactly P(s), as in [33,34], and opacity depends solely on the erasure of unobservable events.
2. *Timed observations*: events carry timestamps or are associated with timing constraints, which allows the intruder to refine inference based on temporal patterns and can endanger opacity [44,45,46,47].
3. *Delayed observation*: the intruder receives observations subject to communication delays, packet losses, or disorder, as in networked settings [48,49,50,51].
4. *Probabilistic observations*: the observation process is subject to random erasures or channel effects; the observer receives events according to some probability law, as in probabilistic opacity frameworks [52,53].
5. *Asymmetric observations*: different agents (e.g., user/defender and intruder) see different subsets of events, with no inclusion relation between them, as in [65,151].

These observation structures enrich the classical projection model and reveal how communication, sensing imperfections, and timing can significantly affect the feasibility and robustness of opacity.

### 6.3. Intruder Knowledge Representation

Once a plant model and an observation structure are fixed, the knowledge representation formalizes how the intruder interprets observations, updates its internal belief about the system’s possible states or behaviors, and decides whether a secret conclusion can be drawn [1,150].

#### 6.3.1. Knowledge as Set-Valued State Estimation

In the classical DES literature, the intruder is modeled as a passive state estimator [33,34,35,37,40]. Given an observation $\alpha \in \Sigma_{o}^{*}$, the estimate of the intruder is

$$E\left(\alpha \right)=\{\;x\in X|\exists s\in L\left(G\right):\delta \left(x_{0},s\right)=x,\;P\left(s\right)=\alpha \;\},$$

which contains all states of the plant that are consistent with at least one execution whose projected observation matches α. This set-valued representation is foundational to state-based opacity notions: opacity typically requires that E(α) always contains at least one nonsecret state whenever secret states are possible.

#### 6.3.2. Knowledge via Language Semantics

In language-based opacity, the intruder reasons about execution strings rather than internal states. For an observable sequence $\alpha \in \Sigma_{o}^{*}$, the intruder considers the set of all system strings whose projection equals α,

$$L_{G}\left(\alpha \right)=\left\{\;\lambda \in L\left(G\right)|P\left(\lambda \right)=\alpha \;\right\}.$$

A secret is specified as a subset $L_{s}\subseteq L\left(G\right)$, and opacity requires that after any observation α, the intruder cannot conclude that all strings in $L_{G}\left(\alpha \right)$ lie in $L_{s}$. This language-based perspective is used, for example, in runtime validation and enforcement of various opacity properties [150,152].

#### 6.3.3. Other Knowledge Models

In stochastic DES representations, the intruder maintains a probability distribution (belief vector) over the state space, which is updated using Bayes-style filtering conditioned on observed events. Such probabilistic knowledge models are appropriate when transitions or observations are inherently random, and they underlie probabilistic opacity formulations [52,53].

Timed DESs introduce observations with timestamps or timing constraints. In these models, intruder knowledge is represented over time-region or marking-class structures, allowing executions to be distinguished based on their temporal patterns [44,45,46,47].

More recent works consider epistemic extensions, where the secret concerns what an agent knows about another agent’s knowledge, leading to notions such as high-order opacity and pre-opacity [74,153]. In fuzzy and approximate settings, knowledge may be represented by fuzzy sets or metric neighborhoods rather than crisp subsets of states [54,55].

Opacity notions in later Section formalize this requirement by specifying, for each type of knowledge representation, the conditions under which the intruder’s knowledge remains sufficiently ambiguous to preserve the secrecy specification.

**Figure 5:**
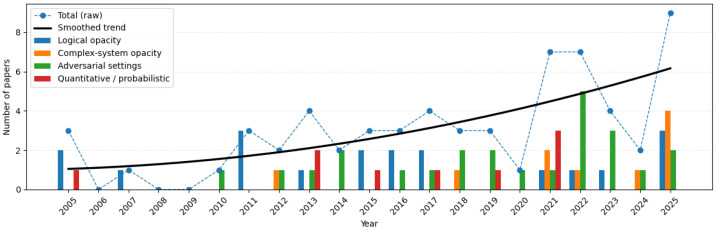
Evolution of opacity research across four complementary directions.

To structure the developments in the literature, we classify existing work into four complementary categories that reflect the evolution of the field, as shown in Figure 5. The first category concerns *pure logical opacity notions*, where opacity is defined as a qualitative indistinguishability property on automata or Petri nets (e.g., CSO, ISO, IFO, *K*-step opacity, and language-based opacity). The second category captures *opacity in complex system contexts*, where classical definitions are extended to networked DESs, distributed or modular systems, and timed models. The third category comprises *adversarial and strategic settings*, in which opacity is treated as a security problem involving an active intruder. The final category concerns *quantitative and probabilistic opacity*, including probabilistic, fuzzy, approximate, and metric-based opacity, as well as quantitative leakage measures such as exposure time and revelation time.

This four-way classification illuminates the trajectory of opacity research: early work focused on defining and verifying qualitative notions, whereas recent work increasingly addresses complex architectures, adversarial interactions, and quantitative assessments of information leakage.

In cyber–physical and industrial control systems, opacity captures fundamental security and privacy requirements, such as preventing external observers or attackers from inferring system operating modes, production schedules, fault states, or control strategies. For example, in ICS and smart grid applications, the disclosure of internal states or event sequences may reveal system vulnerabilities, facilitate targeted attacks, or compromise operational confidentiality. Opacity notions therefore formalize information-flow constraints that arise naturally in secure monitoring, intrusion-aware supervision, and privacy-preserving control architectures.

## 7. Opacity Notions

Opacity formalizes the requirement that an external intruder, equipped with the partial observations and knowledge representations introduced earlier, must remain unable to determine whether a designated secret behavior has occurred. A variety of opacity notions have been developed, depending on whether the secret is specified as a state, a set of executions, a temporal property, or a probabilistic or epistemic condition. This section reviews the main notions in a unified manner and groups them into *logical* and *non-logical* opacity.

### 7.1. Logical Opacity

Logical opacity refers to secrecy properties defined purely through set-based indistinguishability under the intruder’s observation mapping. Such properties are qualitative: the system either satisfies the opacity condition or it does not, and no intermediate degrees of secrecy are considered. These notions form the classical foundation of the field and underpin most early work on opacity.

#### 7.1.1. Language-Based Opacity (LBO)

Language-based opacity concerns secrecy of entire behaviors. Given a secret language $L_{s}\subseteq L\left(G\right)$, the intruder observes an observation word $\alpha \in \Sigma_{o}^{*}$ and considers all system executions whose projection equals α. Opacity holds if the intruder never encounters an observation whose only possible preimages are secret executions [34,150,152]. This notion is central in runtime monitoring and trace-based validation tasks.

#### 7.1.2. Current-State Opacity (CSO)

Current-state opacity focuses on the plant’s possible states after an observed execution. Let $X_{s}\subseteq X$ denote the set of secret states. After observing α, the intruder forms a state estimate E(α) consisting of all states reachable by some execution with projection α. CSO requires that whenever the system actually reaches only secret states, the corresponding estimate E(α) must still include at least one nonsecret state. Thus, the intruder can never conclude, based on observation alone, that the current state lies entirely inside the secret region. CSO is the most extensively studied opacity notion [33,37,41,42,43,67,68].

#### 7.1.3. Initial-State Opacity (ISO)

Initial-state opacity protects confidentiality of the initial configuration. When different initial states have indistinguishable observable behavior, the intruder must never be able to infer that the system began in a secret initial state, even after observing arbitrarily long executions [36]. This notion is particularly relevant when the initial condition encodes private information.

#### 7.1.4. Initial-and-Final-State Opacity (IFSO)

Initial-and-final-state opacity generalizes ISO by simultaneously requiring ambiguity about both the system’s starting and ending state [154]. Even after observing a complete execution, the intruder must be unable to determine whether the system started from a secret initial state or terminated in a secret final state. Recent work refines this notion by introducing stronger temporal variants that enforce secrecy at all intermediate points [39,40].

#### 7.1.5. *K*-Step and Infinite-Step Opacity

*K*-step opacity requires ambiguity about whether the system visited a secret state within the last *K* events prior to the current observation [35]. Infinite-step opacity strengthens this requirement so that the intruder must never become certain about any past secret visit, regardless of how long ago it occurred [155]. These notions impose temporal depth on secrecy and are widely used in attack detection and privacy-preserving diagnosis.

#### 7.1.6. High-Order and Epistemic Opacity

Epistemic extensions of opacity capture settings where the secret concerns not only the system state but also what one agent knows about another agent’s knowledge. High-order opacity ensures that the intruder cannot deduce that another agent has inferred the secret [153]. Pre-opacity protects future intentions rather than past or present behavior, requiring the intruder to remain uncertain about whether the system is guaranteed to reach a secret state in all future continuations [74]. These extensions connect opacity with epistemic logic and multi-agent reasoning.

### 7.2. Non-Logical Opacity Notions

Non-logical opacity generalizes the classical notions by incorporating timing, probability, fuzziness, communication effects, or continuous leakage metrics. Rather than a Boolean yes/no condition, secrecy is evaluated in terms of how robustly the system maintains ambiguity under realistic conditions.

#### 7.2.1. Timed Opacity

In timed automata/Petri nets, timestamps and timing constraints can enable the intruder to distinguish executions that are observationally identical in the untimed sense. Timed opacity therefore requires preserving ambiguity in both the discrete event sequence and the temporal evolution [44,45,46,47]. This has led to notions such as opaque time, time-bounded opacity, and timed current-state opacity.

#### 7.2.2. Probabilistic Opacity

In probabilistic opacity, the intruder maintains a belief distribution over the state space. Opacity requires that the posterior probability assigned to secret behavior remains below a specified threshold or that the intruder never reaches certainty about the secret. Additional continuous leakage metrics, such as exposure or revelation time, quantify the rate at which information accumulates [44,52,53].

#### 7.2.3. Fuzzy and Approximate Opacity

Fuzzy and approximate opacity arise when states or observations have graded membership or metric uncertainty rather than crisp boundaries. Opacity is expressed as an approximate indistinguishability requirement: the observed behavior must remain sufficiently close (in a fuzzy or metric sense) to at least one nonsecret behavior [54,55]. These notions are motivated by noisy sensors, approximate measurements, or hybrid cyber–physical settings.

**Remark** **1.** *Logical opacity demands* binary *secrecy: the intruder must never be able to rule out all nonsecret alternatives. Non-logical opacity broadens this framework by allowing secrecy to be* quantified *(probabilistic or fuzzy),* distorted *(due to timing or network effects), or* epistemic *(involving multi-agent knowledge). Despite these differences, all opacity notions share the same essential requirement: every observation must preserve enough ambiguity to prevent the intruder from conclusively inferring the secret.*

From a sensing standpoint, opacity formalizes the requirement that sensor observations, logs, or communicated measurements should not allow an external observer to infer sensitive system states or execution histories. In this sense, opacity captures information-flow security constraints induced by sensing architectures. Opacity violations thus correspond to information leakage through sensor exposure, while opacity enforcement mechanisms aim to regulate or distort sensor-visible behavior to preserve confidentiality.

## 8. Opacity Verification Approaches

Opacity verification has evolved into a technically rich research area spanning automata, Petri nets, timed models, and stochastic or fuzzy formalisms. Despite the diversity of these models, the core question is always the same: whether an external observer, based solely on its partial observations, can uniquely infer that a secret state or behavior has occurred. Over the past two decades, verification techniques have progressed from classical observer constructions for finite automata to sophisticated symbolic abstractions for concurrent and timed systems and to quantitative frameworks that measure information leakage. This section reviews these developments in a unified manner.

### 8.1. Opacity Verification in Automata

Finite state automata remain the foundational setting for opacity verification, owing to their regular-language structure and compatibility with observer-based reasoning. Most classical opacity notions, including CSO, ISO, and LBO, were first formalized in this framework.

The verification of CSO was established in [33] using an observer automaton that tracks state-estimate evolution under partial observation. A violation occurs when an estimate becomes a subset of the secret states. While the observer construction is polynomial in the number of transitions, its size is exponential in the number of states due to the subset construction.

LBO was studied extensively by Lin [34], which provided verification methods for strong and weak variants by constructing synchronized products that compare strings sharing the same observation. These constructions also incur exponential complexity.

Saboori and Hadjicostis later developed the verification framework for ISO [36]. Their initial-state estimator aggregates all initial states consistent with an observed word, and ISO is violated when an estimate contains no nonsecret initial state. This approach leads to PSPACE-complete complexity even for deterministic automata.

Temporal opacity extensions required more elaborate estimators. *K*-step opacity and infinite-step opacity, introduced in [35,155], rely on estimators that track both current and past states, resulting in doubly exponential worst-case complexity. A significant breakthrough came with the two-way observer of Yin and Lafortune [156], which separates forward and backward information propagation and yields far more scalable verification for *K*-step and infinite-step opacity.

Across these developments, the common technique is the construction of an observer-like structure that encodes all states consistent with a given observation. The challenge lies in controlling the exponential growth of these structures while preserving completeness.

### 8.2. Opacity Verification Using Petri Nets

Petri nets provide a natural modeling formalism for concurrent and distributed DESs, where multiple transitions may fire independently. This concurrency makes traditional observer constructions infeasible, and opacity verification methods have therefore diverged considerably from their automata counterparts.

Opacity in Petri nets was first addressed by Bryans et al. [157], which established that while certain opacity notions are decidable for bounded nets, opacity becomes undecidable in general. This motivated the development of symbolic abstractions that avoid explicit reachability-graph construction.

Tong et al. adopted the basis reachability graph (BRG) [41,158,159] in opacity problems. The BRG compactly represents reachable markings using equivalence classes based on minimal explanations of unobservable transitions. This symbolic abstraction supports verification of CSO and ISO without constructing the full reachability graph such that scalability is improved. For LBO, Tong et al. [160] developed the verifier-net method, which avoids enumerating all firing sequences and yields efficient verification for bounded nets whose unobservable subnet is acyclic.

Beyond BRG-based approaches, Basile and Tommasi [161] proposed an algebraic method for language-based opacity based on integer linear programming (ILP). This method applies even to unbounded nets, eliminating the need for reachability analysis and instead checking opacity through structural constraints encoded as linear inequalities.

Cong et al. further extended opacity verification to online settings [162,163], where ILP queries are evaluated at runtime, enabling on-the-fly CSO and ISO verification. Recent work also considers decentralized and distributed observers, allowing multiple intruders to infer secrecy collaboratively.

Overall, Petri-net verification emphasizes symbolic reachability, structure-based abstractions, and algebraic characterizations—techniques needed to handle concurrency and large state spaces.

### 8.3. Quantitative Verification

Binary opacity verification is often too restrictive in systems where partial information leakage is unavoidable or acceptable up to a threshold. This led to the development of quantitative opacity measures that evaluate secrecy along probabilistic, fuzzy, or metric dimensions.

Foundational work by Lakhnech and Mazaré introduced probabilistic opacity and cryptographic interpretations of secrecy [164]. Bryans et al. [165] later formalized probabilistic opacity in transition systems, laying the groundwork for quantitative leakage analysis.

A major advance came from Bérard et al. [166], which introduced the dual measures of Liberal Probabilistic Opacity (LPO); the probability that the intruder can confirm the secret; and Restrictive Probabilistic Opacity (RPO), the expected uncertainty preserved about the secret. These metrics allow fine-grained assessment of information leakage.

Saboori and Hadjicostis defined probabilistic current-state opacity and developed Markov chain estimators [167]. Keroglou and Hadjicostis extended this to probabilistic initial-state opacity and provided a polynomial-time algorithm for a class of probabilistic systems [52,168]. Yin et al. [53] introduced infinite-step and K-step probabilistic opacity using belief-state abstractions.

Fuzzy opacity [54] generalizes secrecy to settings with graded membership, requiring that the possibility degree of secret behaviors never dominates that of nonsecret behaviors. Approximate opacity [55] uses metrics on state trajectories to ensure that observations remain “close enough” to nonsecret behavior in cyber–physical systems subject to noise.

Timing further complicates opacity verification. Temporal information can distinguish executions that are otherwise observationally identical, requiring region graphs, zone graphs, or marking-class graphs for analysis. Early timed opacity notions were refined in [44], which introduced exposure and revelation time metrics for timed stochastic DESs. Recent work in networked systems examines opacity under communication delays, losses, and reordering [49]. Time LPNs [45,46,47] support fine-grained reasoning about timing intervals and have inspired new verification procedures based on time-expanded state-space analysis.

Across all quantitative frameworks, verification reduces to analyzing the intruder’s evolving belief or possibility distribution. While conceptually straightforward, these belief spaces are often continuous or high-dimensional, making abstraction and convexity tools essential.

In summary, opacity verification has progressed from observer-based automata techniques to symbolic Petri-net abstractions and quantitative frameworks for timed, probabilistic, and fuzzy systems. Despite major advances, the computational complexity of verifying opacity remains high (often PSPACE-hard or exponential), which highlights the need for scalable abstractions, compositional reasoning, and data-driven verification techniques.

## 9. Opacity Enforcement Approaches

Opacity enforcement concerns modifying either the system’s behavior or the information revealed to the intruder so that, under the given observation structure and knowledge representation, the secret cannot be deduced. Whereas verification asks whether the plant already satisfies the opacity requirement, enforcement aims to transform the system without violating admissibility or correctness constraints so that opacity holds for all executions consistent with the enforced behavior. Enforcement strategies mainly differ according to whether they modify the information revealed to the intruder or constrain the evolution of the system through supervisory control. Across these diverse approaches, the unifying goal is to restore or preserve indistinguishability whenever the system’s natural behavior would otherwise expose the secret.

### 9.1. Channel-Based Enforcement: Edit and Insertion Mechanisms

**Figure 6:**
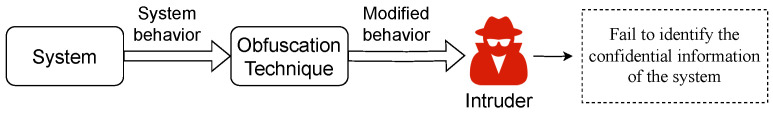
Obfuscation-based enforcement architecture acting on the observation channel.

One of the most extensively studied enforcement paradigms is based on *edit functions*, *insertion functions*, and related obfuscation mechanisms that act directly on the observation channel between the plant and the intruder (shown in Figure 6). The key idea is that the intruder no longer sees the observations generated by the system, but rather a modified observation outputted by an obfuscation mechanism. This line of research originates from the seminal work of Wu and Lafortune [56], who introduced insertion functions to enforce opacity by augmenting observable traces with fictitious events so that every secret-revealing observation becomes indistinguishable from a nonsecret alternative. Their subsequent development of optimal insertion functions incorporated cost criteria and showed how to synthesize minimally disruptive policies that preserve opacity while adhering to constraints on the allowed insertions [57]. Edit functions, introduced in [169], generalize this idea by allowing insertion, deletion, and substitution.

Building on this foundation, Ji et al. [170] further investigated scenarios in which insertion functions may be known to the intruder. Moreover, they introduced *nondeterministic* publicly known edit functions [58], in which the additional nondeterminism enlarges the set of edited behaviors and can make opacity enforcement feasible in cases where deterministic insertion would fail. Keroglou and Lafortune proposed *embedded* insertion functions, where the enforcer is integrated within the plant model and must satisfy structural constraints on when and how insertions can occur [60]. These ideas were further refined in the work of Li et al., who developed *extended* insertion functions capable of handling constraints on the inserted language and later proposed modification functions that unify insertion and deletion within a single enforcement framework [61,62,66].

To address scalability, Mohajerani et al. introduced a compositional and abstraction-based synthesis method for edit functions [59], which enables enforcement in large-scale composed systems by working on abstract models and refining the resulting editors back to the original plant. More recently, Liu et al. [63,64] investigated greedy synthesis of privately and publicly known insertion functions and proposed improved constructions for nondeterministic publicly known editors, focusing on complexity reduction and practical implementability. Duan et al. introduced event concealment and concealability enforcement, which address opacity at the level of events rather than states and show how obfuscation mechanisms can be used to enforce event-level secrecy in an efficient manner [72].

A central challenge throughout this line of work is to guarantee that the editing process is realizable and does not introduce blocking or inconsistencies in the modified observation stream. This motivated the synthesis of edit mechanisms as finite automata whose accepted language matches the set of all edited observations consistent with some plant execution, so that every edit corresponds to a feasible continuation of at least one system trajectory. More recent work has examined enforcement under uncertain or degraded observation on the editor side, where the edit mechanism operates with its own (possibly incomparable) observation structure relative to the intruder [65]. In such settings, the editor must guarantee opacity even though it does not fully know what the intruder sees.

Overall, channel-based enforcement is attractive because it preserves the plant’s behavior, requires no control authority over events, and is compatible with the original systems. Its limiting factor is the need for realizability and linguistic consistency between the edited output and the plant’s observable language.

### 9.2. Control-Based Enforcement: Supervisory and Structural Approaches

**Figure 7:**
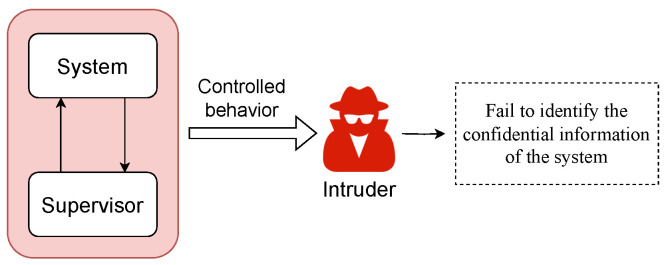
Opacity-enforcing supervisory control architecture.

A complementary paradigm constrains the plant execution rather than modifying observations. In this view, opacity enforcement is cast as a supervisory control problem (shown in Figure 7): the supervisor observes the system (under its own partial observation) and disables selected controllable events so that no secret-revealing behavior remains reachable.

The earliest systematic treatment of this idea appears in the work of Dubreil et al. [67], who formulated opacity enforcement as a supervisory control problem and characterized conditions for the existence of supervisors enforcing language-based opacity. Saboori and Hadjicostis [68] subsequently developed a state-estimator-based synthesis method that constructs opacity-enforcing supervisors from an observer-like structure, thereby connecting opacity enforcement with classical DES supervision.

Later contributions extended these ideas to decentralized and more complex architectures. Tong et al. considered decentralized opacity enforcement, where multiple local supervisors with limited observation and control must cooperate to enforce a global opacity specification [69]. Xie et al. introduced *nondeterministic* supervisors for opacity enforcement, showing that nondeterministic control laws can enlarge the class of enforceable opacity requirements compared to deterministic supervisors [70]. Moulton et al. proposed using subobservers to synthesize opacity-enforcing supervisors, emphasizing the reduction in state-space explosion by working on compressed observer structures [71].

Petri-net and distributed DES models require analogous but structurally richer control-based enforcement strategies. In labeled and time LPNs, secret-revealing markings may arise due to independent or concurrent firings, and opacity-preserving control laws are typically derived from symbolic structures such as labeled Petri-net observers, verifier nets, basis reachability graphs, or marking-class graphs [41,42,43,45,46,47].

In distributed or modular systems, different components may have distinct observability and controllability profiles, motivating decentralized or cooperative opacity enforcement schemes. Paoli and Lin studied decentralized opacity for DESs and highlighted fundamental limitations when local observers lack sufficient information [73].

In networked DESs, the communication channel itself becomes an adversarial element: delays, losses, or reordering can create or eliminate ambiguity. Control-based enforcement may therefore need to regulate not only plant-level transitions but also communication behavior, for instance by shaping the timing or acknowledgment patterns of transmitted events [49]. Recent work on online opacity verification and enforcement for networked Petri nets and automation systems further develops this perspective, with Li et al. proposing online opacity verification algorithms for networked Petri-net models that naturally suggest online enforcement mechanisms [50]. In such frameworks, enforcement decisions are updated as events are produced and transmitted, ensuring that opacity is maintained in real time despite channel uncertainties and network dynamics.

In summary, control-based enforcement is powerful when control authority exists, and it aligns well with standard DES supervision. Its limitations stem from controllability restrictions, decentralized information structures, and scalability of estimator-based synthesis.

### 9.3. Comparison Between Channel-Based and Control-Based Enforcement

Channel-based and control-based opacity enforcement represents fundamentally different strategies for preserving confidentiality under partial observation. Control-based enforcement relies on supervisory control to restrict or modify system behavior so that opacity is maintained. Such an approach provides strong formal guarantees and integrates naturally with existing control architectures but may reduce system permissiveness or performance due to behavior disabling or restriction.

In contrast, channel-based enforcement mechanisms, such as edit and insertion functions, aim to preserve the original plant behavior by manipulating the information revealed through observation channels. These methods are particularly attractive when direct control over the system is limited or undesirable and when confidentiality must be enforced without altering physical behavior. However, channel-based approaches introduce additional challenges related to realizability, boundedness, and computational complexity of obfuscation mechanisms, and often rely on assumptions about communication capabilities and attacker models.

From a practical standpoint, control-based enforcement is well suited to settings where supervisory authority is available and behavioral modification is acceptable, while channel-based enforcement is more appropriate in monitoring or auditing contexts where behavior preservation is critical. In many applications, hybrid approaches combining control and channel manipulation may offer a favorable trade-off between confidentiality, performance, and implementability.

### 9.4. Enforcement for Non-Logical Opacity

Non-logical opacity introduces probabilistic, fuzzy, metric, or timed dimensions, for which classical editing or supervisory control techniques must be adapted or generalized.

Probabilistic opacity requires that the intruder’s posterior probability of secret behavior remain below a desired threshold. Foundational work by Keroglou and Hadjicostis [52] and later by Yin et al. [53] showed how belief distributions propagate in stochastic DESs, leading to infinite-step and *K*-step probabilistic opacity definitions. Enforcement may modify transition probabilities, randomize event occurrences, or introduce probabilistic editing policies so that the probability of revealing the secret remains acceptably low.

In fuzzy opacity [54], states and observations have degrees of membership. Enforcement must preserve the ordering of secret versus nonsecret possibility levels. Approximate opacity [55] replaces crisp equality with metric closeness: the enforcer ensures that every observed behavior lies within an ε-ball of some nonsecret behavior, relevant in CPSs subject to noise.

Exposure and revelation metrics [44] quantify how long secrets remain protected before becoming inferable. Enforcement strategies may slow down secret-related transitions or synchronize timing to reduce distinguishability. Network-induced uncertainty, including delays and reordering, adds another layer where enforcement regulates transmission timing or acknowledgment patterns.

Table 2 summarizes the key differences between observer-based and structural or algebraic approaches to opacity verification and enforcement. Observer-based methods offer a conceptually unified framework for reasoning about intruder knowledge evolution, but often suffer from severe state-space explosion. Structural and algebraic techniques exploit concurrency and system structure to improve scalability, particularly for Petri net models, at the cost of stronger modeling assumptions. The table also clarifies the complementary roles of control-based and channel-based enforcement: the former modifies system behavior to ensure opacity, while the latter preserves behavior by manipulating information exposed through observation channels.

## 10. Diagnosis vs. Opacity

### 10.1. Diagnosis and Opacity Analysis

Fault diagnosis and opacity are both concerned with reasoning about hidden information in a partially observed DES. In fault diagnosis, the goal is to determine, from observable behavior, whether an unobservable fault has occurred. In opacity, the aim is to prevent an external observer from determining whether a confidential behavior has occurred. Thus, although the motivations differ, the two problems share a common informational structure.

This connection becomes evident when comparing their core definitions. A system is *diagnosable* if every faulty execution eventually becomes distinguishable from all nonfaulty ones; that is, the diagnoser’s estimate must ultimately collapse to the fault set. Opacity requires the opposite evolution: every secret execution must remain indistinguishable from at least one nonsecret execution, so the intruder’s estimate must *never* collapse to the secret set.

Despite this inverted objective, the technical machinery is largely shared. Both problems rely on state estimators that track the set of states consistent with the observed sequence, and both verify conditions on the reachability of designated subsets of this estimate. Observer automata, twin-plant constructions, and symbolic Petri-net abstractions (e.g., BRGs ad verifier nets) appear in both diagnosis and opacity analysis. In probabilistic settings, the same belief-update mechanisms underpin probabilistic diagnosability and probabilistic opacity.

The duality extends naturally to enforcement. Fault-tolerant control seeks to guarantee that faults can be detected or mitigated; opacity-enforcing mechanisms seek to prevent disclosure of secret-revealing behaviors. In both cases, feasibility depends on how controllability and observability constraints influence the evolution of the observer’s knowledge.

Conceptually, diagnosis and opacity can therefore be viewed as complementary information-flow problems. Diagnosis requires that the system become sufficiently informative for an observer to infer the fault, while opacity requires that the system remains sufficiently ambiguous to hide the secret. By presenting them side by side, their shared methodological roots and opposing operational objectives become clear, highlighting that both are instances of reasoning about knowledge evolution in partially observed DESs.

Advanced constructs such as K-step opacity, basis reachability graphs, and verifier nets should be interpreted as scalability-enabling abstractions rather than purely theoretical refinements. In real industrial systems, concurrency, timing constraints, and large or unbounded state spaces make explicit state enumeration infeasible. Symbolic structures like basis reachability graphs and verifier nets allow opacity and diagnosability properties to be verified and enforced without constructing the full reachability graph, thereby enabling analysis of complex manufacturing systems, transportation networks, and networked automation architectures. Similarly, K-step opacity reflects practical requirements where only recent system behavior is considered sensitive, aligning with sliding-window monitoring and limited-memory intrusion detection mechanisms used in practice.

While fault diagnosis and opacity originate from different application goals, their verification and enforcement procedures rely on closely related observer-based constructions. To complement the conceptual discussion, Table 3 summarizes the main technical correspondence between diagnosis and opacity by aligning their main analysis tasks and associated constructs. This comparison highlights that many mathematical tools are structurally similar yet are used for opposite purposes: diagnosis aims to eliminate ambiguity about faults, whereas opacity aims to preserve ambiguity about secrets.

### 10.2. Computational Complexity and Scalability Considerations

A recurring challenge in both fault diagnosis and opacity analysis is the combinatorial explosion caused by partial observation, concurrency, and temporal extensions. While many verification and enforcement problems are known to be PSPACE-complete or exponential in the worst case, the practical applicability of a method depends critically on the chosen modeling formalism, abstraction strategy, and structural assumptions. In this subsection, we provide a comparative perspective on the computational characteristics of representative approaches, with the aim of clarifying which methods are suitable for medium-scale systems and which primarily serve as theoretical benchmarks.

Observer-based methods for automata-based models, while conceptually simple, suffer from exponential state-space growth due to subset construction and are typically limited to small or moderately sized systems. Verifier-based and twin-plant approaches reduce complexity by avoiding full observer construction and are often applicable to medium-scale automata under reasonable observability assumptions.

In Petri net models, reachability-graph-based techniques quickly become infeasible due to unboundedness and concurrency. Symbolic abstractions such as basis reachability graphs and verifier nets significantly improve scalability by exploiting structural properties of unobservable subnets and are among the few approaches applicable to medium-scale concurrent systems

Algebraic and ILP-based methods avoid explicit state enumeration and are therefore well suited for large or unbounded Petri nets; however, they typically shift complexity to solving integer programs, which may affect online applicability.

Quantitative opacity and timed extensions introduce additional sources of complexity, often leading to doubly exponential constructions. These methods are primarily applicable to small systems or offline analysis, although abstraction and compositional techniques can mitigate complexity in specific cases.

Moreover, Table 4 provides a comparative overview of verification and enforcement methods, highlighting which approaches are primarily of theoretical interest and which are commonly applicable to medium-scale industrial systems under reasonable modeling assumptions.

## 11. Future Directions and Open Problems

**Figure 8:**
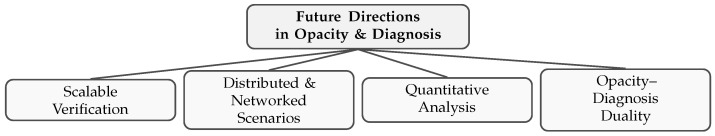
A compact roadmap of major research directions in opacity and diagnosis.

As DESs increasingly underpin cyber–physical systems, communication infrastructures, and distributed autonomous platforms, understanding what observers can infer and how such inference can be constrained becomes both more essential and more difficult. This section highlights several key research directions (shown in Figure 8) where substantial opportunities remain across modeling, verification, and enforcement.

### 11.1. Scalability and Complexity

Even with significant advances in observer reduction, symbolic Petri-net abstractions, and timed DES approximations [41,42,43,44,47], diagnosis/opacity verification remains computationally intensive for systems with large state spaces. Knowledge estimates may grow exponentially, and product-based verification may be infeasible in practice. Promising directions include compositional and concurrency-aware abstractions, on-the-fly search, and learning-based reductions. However, a unifying verification theory that ensures both soundness and tractability across heterogeneous models is still lacking.

### 11.2. Decentralized and Distributed Settings

Many real systems rely on asynchronous communication and heterogeneous sensing. Editors and supervisors may not know precisely what other agents observe, and opacity under incomparable or asymmetric observation structures remains largely open [65]. Online enforcement becomes particularly difficult when decisions must be taken without knowledge of current channel delays, losses, or reorderings [48,50]. Understanding how network-induced uncertainty interacts with opacity-enforcing mechanisms calls for new theoretical tools and architectural designs capable of maintaining secrecy without degrading performance.

### 11.3. Non-Logical Diagnosis/Opacity Frameworks

While quantitative verification methods exist [52,53,54], general and scalable *enforcement* mechanisms remain underdeveloped. A rigorous theory for shaping belief evolution, manipulating possibility distributions, or controlling epistemic relations is still missing. Epistemic opacity, in particular, introduces multi-agent interactions where the secret may concern not only system states but also what one observer knows about another’s knowledge. Extending diagnosis/opacity to strategic or adversarial multi-agent scenarios is a promising frontier connecting DES with epistemic logic, game theory, and distributed systems.

### 11.4. Hybrid Model-Based and Data-Driven Approaches

Another interesting research direction concerns the integration of logical DES frameworks with machine learning and data-driven techniques. Learning-based methods may help address model uncertainty, scalability, and adaptivity, while formal DES-based approaches provide correctness guarantees. Open questions include how to combine learning with diagnosability and opacity-related enforcement analysis, and how to ensure interpretability in hybrid model-based and data-driven architectures.

In summary, while the foundational theory of opacity and diagnosis is well-established, their application to autonomous, networked, uncertain, and data-driven systems raises significant new challenges. Progress will require new models, scalable algorithms, enriched information structures, and interdisciplinary methods drawing from control theory, computer science, information theory, and game theory. Inference under partial observation remains at the core of these efforts, ensuring that secrecy can be preserved when desired and revealed when necessary.

## 12. Conclusions

This survey has presented a comprehensive overview of recent advances in fault diagnosis and opacity analysis for discrete event systems. We reviewed classical foundations together with modern developments in diagnosability verification and enforcement, covering automata, Petri net, and extended DES modeling frameworks. Special attention was given to challenges introduced by communication losses, delays, distributed architectures, timing constraints, and various classes of cyber attacks. Parallel to fault diagnosis, we examined opacity verification and enforcement techniques, highlighting their conceptual links to information security, privacy protection, and critical state confidentiality.

Despite significant progress, important challenges remain. Key directions include improving the scalability of verification and enforcement algorithms; integrating diagnosis and opacity considerations within unified frameworks; and designing robust, attack-resilient methodologies suitable for increasingly networked and adversarial environments. By consolidating the current state of the art and identifying open problems, this survey aims to provide a valuable reference for researchers and practitioners and to help shape future work on resilient and secure DESs.

## Figures and Tables

**Table 1:** Comparative analysis of fault diagnosis frameworks and methods.

Aspect	Automata-Based Diagnosis	Petri Net-Based Diagnosis	Main Advantages	Main Limitations/Trade-Offs
Modeling paradigm	Finite-state, sequential event abstraction	Concurrent, distributed, resource-sharing models	Conceptual simplicity (automata); natural concurrency modeling (PNs)	Limited concurrency (automata); higher structural complexity (PNs)
Diagnosability verification	Diagnoser, verifier, twin-plant constructions	Basis reachability graph, verifier nets, ILP-based analysis	Well-established theory (automata); scalable symbolic abstractions (PNs)	Exponential observer growth (automata); PSPACE-complete or undecidable cases (PNs)
Online fault diagnosis	State estimation via observers/diagnosers	Marking estimation via ILP or structure-based observers	Clear runtime monitoring logic	Online ILP or structural checks may be computationally demanding
Unbounded systems	Generally not applicable	Explicitly addressed via structural and algebraic techniques	Enables modeling of realistic industrial systems	Possible loss of precision or conservativeness
Enforcement mechanisms	Supervisory control, sensor relabeling, sensor activation	Control places, structural constraints	Direct control over system evolution	Reduced permissiveness or throughput
Robustness to sensing imperfections	Extensions for delays, losses, and attacks	Networked and attack-resilient diagnosis frameworks	Captures realistic sensing conditions	Increased modeling and verification complexity

**Table 2:** Comparative analysis of opacity verification and enforcement paradigms.

Aspect	Observer-Based Approaches	Structural/Algebraic Approaches	Advantages	Limitations
Opacity verification	Observers, two-way observers, language-based checks	BRG, verifier nets, ILP-based verification	Unified belief-based framework	Exponential complexity; undecidability in general PNs
Representation of concurrency	Implicit (state-space expansion)	Explicit via PN structure	Accurate modeling of concurrent systems	Requires structural assumptions
Temporal and quantitative opacity	K-step, infinite-step, timed observers	Marking-class graphs, probabilistic abstractions	Expressive secrecy notions	Doubly exponential or PSPACE-hard complexity
Control-based enforcement	Opacity-enforcing supervisors	Structural supervision in PN models	Strong formal guarantees	Reduced permissiveness; controllability constraints
Channel-based enforcement	Edit, insertion, and deletion functions (automata-based)	Not established for Petri net models	Preserves plant behavior	Limited to sequential models; open problem for concurrent systems
Adversarial modeling	Intruder knowledge via observer states	Structural modeling of attack surfaces	Explicit reasoning about inference	Increased synthesis and verification complexity

**Table 3:** Main technical correspondence between fault diagnosis and opacity analysis.

Task	Diagnosis	Opacity	Key Difference
Verification	Diagnoser, verifier automaton	Observer, detector automaton	Diagnosis seeks certainty of faults; opacity seeks persistent ambiguity
Knowledge structure	State estimate distinguishing faulty vs. non-faulty states	State or language estimate distinguishing secret vs. non-secret behaviors	Similar estimator structure, opposite acceptance condition
Violation condition	Indeterminate cycle	Certain estimate	Ambiguity is undesirable in diagnosis but desirable in opacity
Enforcement mechanism	Supervisor, relabeling function, sensor activation	Insertion/edit function, opacity-enforcing supervisor	Diagnosis increases observability; opacity restricts or distorts it

**Table 4:** Comparison of verification and enforcement approaches in terms of computational complexity and practical scalability.

Approach	Model	Typical Complexity	Scalability	Practical Applicability
Observer/diagnoser	Automata	Exponential	Low–Medium	Small systems
Detector/Verifier	Automata	Polynomial–Exponential	Medium	Medium-scale DES
Basis reachability graph	Petri nets	PSPACE-complete	Medium–High	Concurrent industrial systems
Verifier nets	Petri nets	PSPACE-complete	Medium	Bounded or structured nets
ILP-based methods	Petri nets	NP-hard	Medium–High	Large or unbounded systems
K-step/infinite-step estimator	Automata	Exponential–Doubly exponential	Low	Offline or reduced models
Probabilistic/timed estimator	Stochastic/timed DES	PSPACE-hard	Low–Medium	Small systems, risk analysis

## Data Availability

Enquiries about data availability should be directed to the authors.
